# Magnetotactic bacteria from Pavilion Lake, British Columbia

**DOI:** 10.3389/fmicb.2013.00406

**Published:** 2013-12-20

**Authors:** Zachery Oestreicher, Steven K. Lower, Eric Rees, Dennis A. Bazylinski, Brian H. Lower

**Affiliations:** ^1^College of Science and Engineering, Kanazawa UniversityKanazawa, Japan; ^2^School of Earth Sciences, School of Environment & Natural Resources, The Ohio State UniversityColumbus, OH, USA; ^3^Research and Testing LaboratoryLubbock, TX, USA; ^4^School of Life Sciences, University of Nevada at Las VegasNV, USA

**Keywords:** magnetotactic bacteria, microbialites, transmission electron microscope, magnetite nanoparticles, magnetite, magnetosomes

## Abstract

Pavilion Lake is a slightly alkaline, freshwater lake located in British Columbia, Canada (50°51'N, 121°44'W). It is known for unusual organosedimentary structures, called microbialites that are found along the lake basin. These deposits are complex associations of fossilized microbial communities and detrital- or chemical-sedimentary rocks. During the summer, a sediment sample was collected from near the lake's shore, approximately 25–50 cm below the water surface. Magnetotactic bacteria (MTB) were isolated from this sample using a simple magnetic enrichment protocol. The MTB isolated from Pavilion Lake belonged to the *Alphaproteobacteria* class as determined by nucleotide sequences of 16S rRNA genes. Transmission electron microscopy (TEM) revealed that the bacteria were spirillum-shaped and contained a single chain of cuboctahedral-shaped magnetite (Fe_3_O_4_) crystals that were approximately 40 nm in diameter. This discovery of MTB in Pavilion Lake offers an opportunity to better understand the diversity of MTB habitats, the geobiological function of MTB in unique freshwater ecosystems, and search for magnetofossils contained within the lake's microbialites.

## Introduction

Magnetotactic bacteria (MTB) have been found in a variety of aquatic sediments such as marine environments, freshwater lakes and rivers, hot springs, and brackish waters all over the world (Blakemore, 1975; Moench and Konetzka, 1978; Spring et al., 1994; Bazylinski et al., 1995, 2000; Amann et al., 2007; Lin et al., 2009; Lef èvre et al., 2010, 2011). In these settings, MTB tend to reside in chemically stratified water or sediment at the oxic-anoxic interface. The common feature that is unique to all MTB is their ability to synthesize intracellular membrane-bound crystals of single domain magnetite (Fe_3_O_4_) and/or greigite (Fe_3_S_4_) (Bazylinski et al., 1993, 1995; Lower and Bazylinski, 2013). The magnetosomes provide a torque on the cells that passively aligns them with the Earth's geomagnetic field. This in turn reduces their navigational route from three dimensions to one dimension; shortening the time it takes for cells to navigate to their preferred habitat, the oxic-anoxic interface at the bottom of water bodies (Bazylinski et al., 1995; Frankel et al., 2007; Lower and Bazylinski, 2013).

Magnetite from MTB occurs as a very specific size with well-defined crystal morphology that is chemically pure (Devouard et al., 1998; Faivre et al., 2008). Such minerals are preserved in the rock record as “magnetofossils,” which have been found in Mesozoic rocks, and may extend back as far as the pre-Cambrian (Kirschvink and Chang, 1984; Chang and Kirschvink, 1989; Kopp et al., 2007; Kopp and Kirschvink, 2008). The distinct size, purity and crystallinty of magnetite made by MTB help differentiate it from abiogenic magnetite in the fossil record (Petersen et al., 1986; Chang and Kirschvink, 1989; Devouard et al., 1998; Kobayashi et al., 2006; Kopp and Kirschvink, 2008; Benzerara and Menguy, 2009; Benzerara et al., 2011).

This study investigates uncultured MTB from Pavilion Lake, a deep, slightly alkaline, freshwater lake in British Columbia. This site was selected because it contains large organosedimentary structures called microbialites (Laval et al., 2000). Such structures abound in the fossil record back to nearly 3.5 billion years (Lim et al., 2009) and microbialites, in the form of thrombolites, have been dated as far back as the Proterozoic (Kennard and James, 1986). The MTB that we isolated from Pavilion Lake belonged to the *Alphaproteobacteria* class. Transmission electron microscopy was used to determine the morphology of the cells, and the size and shape of magnetosomes. Scanning transmission electron microscopy was used to determine the chemical composition of the magnetosomes. This is the first time MTB have been described from a microbialite-forming environment. This discovery could be of great value to others interested in potential life forms on other planets or the earliest forms of life on Earth as Pavilion Lake contains microbial fossils in the freshwater microbialites.

## Materials and methods

### Magnetotactic bacteria collection

**Figure 1:**
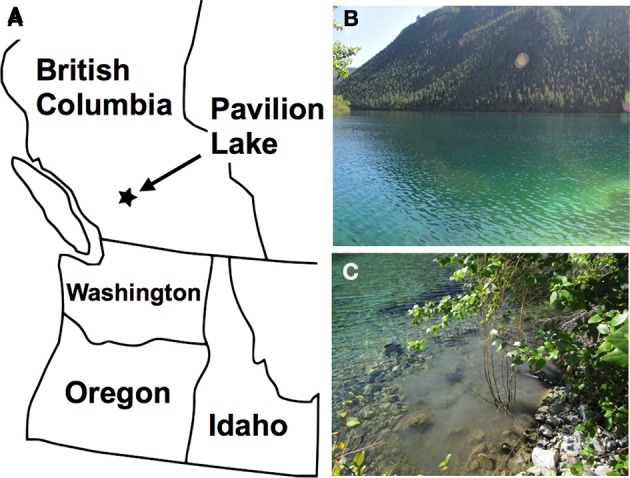
**Map showing the location of Pavilion Lake (A).** Photograph taken in August 2011 showing a portion of Pavilion Lake **(B)**. The water and sediment samples that were used in this study were collected from the northern shore of the lake. **(C)** The disturbed sediment that was visible in the lake after collecting the sample from the shoreline.

Sediment samples were collected from along the shore of Pavilion Lake (Figure 1) in August 2011. Shallow samples of sediment were obtained by scraping the sediment approximately 20–45 cm below the surface of the water with a 1-liter container. The containers contained one-half to three-quarters sediment and the remainder was filled with freshwater from the site. The bottles were sealed and brought back to the laboratory for analysis. In the laboratory, the bottle caps were loosened and stored in a dark at room temperature for up to several weeks.

### Magnetotactic bacteria enrichment

Magnetotactic bacteria were isolated from the sediment following a procedure described previously (Oestreicher et al., 2012). Briefly, the south end of a magnet was placed on the outside of the 1-liter sample container just above the sediment-water interface. The north end of a magnet was placed on the opposite side of the container. After 1 h, the water around the south end of the magnet was extracted with a pipette and placed in a racetrack with a cotton plug at the sealed end (Wolfe et al., 1987). This was repeated 12 times for each sample. The south end of a magnet was placed at the racetrack's sealed end, and the magnetotactic cells were allowed to swim through the cotton barrier for approximately 30 min. The racetrack was taken away from the magnet, the tip snapped off, and the bacteria removed with a syringe. A total of 500–1000 μ L were collected from each sample. The presence of MTB was confirmed by light microscopy on samples prepared by the hanging drop method (Oestreicher et al., 2012).

### Transmission electron microscopy

An aliquot of the enriched cells were placed on a 200 mesh copper grid coated with carbon and formvar (Ted Pella) and analyzed in an FEI Tecnai G^2^ Spirit transmission electron microscope or an FEI Tecnai F20 scanning transmission electron microscope. The accelerating voltage of the G^2^ Spirit was 80 keV with a spot size 2 using the number 2 objective aperture. Images were collected using a Gatan camera and AMT Image Capture software. For the Tecnai F20, an accelerating voltage of 200 keV was used in the high angle annular dark field (HAADF) mode. Crystals inside the cells were analyzed with the energy-dispersive X-ray (EDX) spectrometer on the Tecnai F20 using only a 100 μm condenser aperture. The specimen was tilted 5° toward the EDAX detector that had an ultrathin Moxtek AP3.3 window with an elevation angle of 20°. The size of the cells and the magnetite crystals were analyzed using FIJI software.

### Phylogenetic analysis

Approximately 500 μL of sample collected from the racetrack was used to obtain DNA for phylogenetic analysis. DNA was obtained from the MTB by homogenizing the cells and resuspending them in RLT buffer (Qiagen) with β-mercaptoethanol. A Qiagen DNA kit was used to isolate the DNA. The 16S rRNA genes were amplified by PCR using 28F and 519R primer pairs (TTTGATCNTGGCTCAG and GWNTTACNGCGGCKGCTG, respectively) and a Qiagen hotstart taq mastermix. The DNA was denatured at 95°C for 5 min, followed by 35 cycles at 94°C for 30 s, 54°C for 45 s, 72°C for 60 s. Finally, an extension reaction was performed at 72°C for 10 min.

The amplified DNA was sequenced and analyzed by the Research and Testing Laboratory in Lubbock, Texas. The DNA sequences were aligned using the default settings in MUSCLE (Edgar, 2004a,b). The sequences were compared with reference sequences from NCBI. A phylogenetic tree was generated using these sequences with the sum of branch length = 1.85273957 shown. The tree shown herein is drawn to scale, with branch lengths in the same units as those of the evolutionary distances used to infer the phylogenetic tree (Sneath and Sokal, 1973). The evolutionary distances were computed using the Maximum Composite Likelihood method (Tamura et al., 2004), and are in the units of the number of base substitutions per site. The analysis involved 37 nucleotide sequences. Codon positions included were 1st + 2nd + 3rd + Noncoding. All ambiguous positions were removed for each sequence pair. There were a total of 1711 positions in the final dataset. A total of 16 operational taxonomic units (OTUs) were generated at a clustering identity of 97% (Table S1). Evolutionary analyses were conducted in MEGA5 (Tamura et al., 2011). All sequence data has been deposited in GenBank as SAMN02370231 to SAMN02370259.

## Results

In August 2011, water-sediment samples were collected from Pavilion Lake, British Columbia, Canada (Figure 1). The temperature and pH of the freshwater lake were 20°C and pH 8.3, respectively. This lake is very clear (Figure 1B) with a maximum-recorded depth of 65 m (Laval et al., 2000; Lim et al., 2009). The collected samples were returned to the lab for enrichment of MTB and subsequent analysis of 16S rDNA and examination of the microorganisms by electron microscopy.

**Figure 2:**
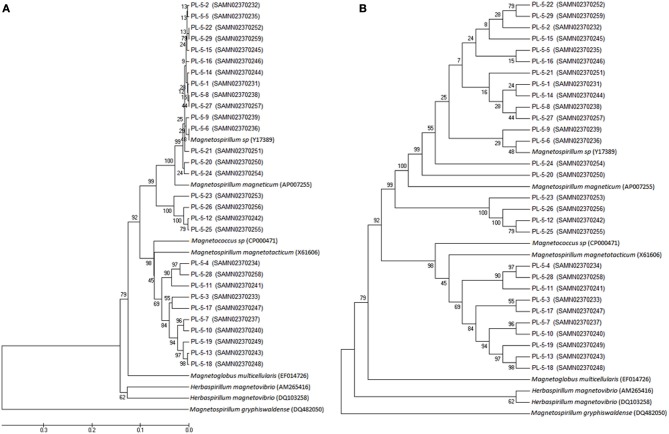
**Phylogenetic tree from twenty-nine 16S rRNA sequences of bacteria isolated from Pavilion Lake, British Columbia. (A)** The evolutionary history for this Original Tree was inferred using the UPGMA method (Sneath and Sokal, 1973). The optimal tree with the sum of branch length = 1.85273957 is shown. The percentage of replicate trees in which the associated taxa clustered together in the bootstrap test (500 replicates) are shown next to the branches (Felsenstein, 1985). The tree is drawn to scale, with branch lengths in the same units as those of the evolutionary distances used to infer the phylogenetic tree. **(B)** The evolutionary history of the Bootstrapped Tree was inferred using the UPGMA method (Sneath and Sokal, 1973). The bootstrap consensus tree inferred from 500 replicates (Felsenstein, 1985) is taken to represent the evolutionary history of the taxa analyzed (Felsenstein, 1985). Branches corresponding to partitions reproduced in less than 50% bootstrap replicates are collapsed. The percentage of replicate trees in which the associated taxa clustered together in the bootstrap test (500 replicates) are shown next to the branches (Felsenstein, 1985).

An aliquot containing enriched MTB from Pavilion Lake was analyzed by 16S sequencing from all the bacteria contained within the enriched sample. The sample contained 29 different 16S rRNA gene sequences named PL-5-1 through PL-5-29. These sequences were compared with sequences in the NCBI nucleotide database in order to construct a phylogenetic tree (Figure 2). An *Alphaproteobacterium* (Genbank accession number DQ482050) was used to root the tree. All 29 of the MTB species isolated from Pavilion Lake were found to group with the *Alphaproteobacteria* (Figure 2).

**Figure 3:**
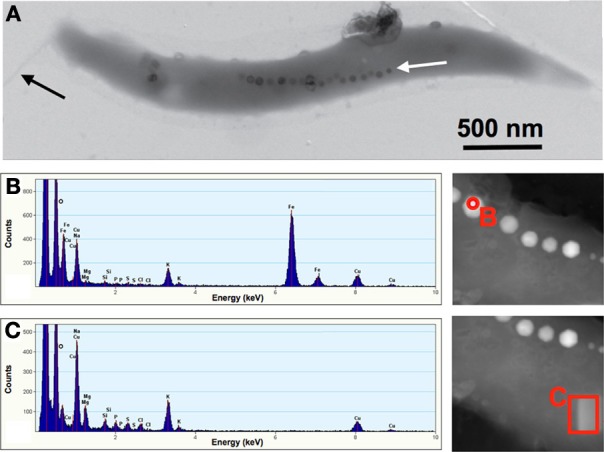
**Spirillum-shaped magnetotactic bacterium from Pavilion Lake, British Columbia, Canada (A).** The cells averaged 2.9 μm long and 0.3 μm wide with a single chain of magnetosomes (white arrow) and a single flagellum (black arrow). Scale bar is 500 nm. The mineral crystals averaged 47 × 44 nm. The nanocrystal marked with a red circle was selected for EDX spectra shown in **(B)**, which shows that the crystals were made of iron and oxygen (copper peak was due to the support grid). The part of the bacterial cell marked with a red rectangle was selected for the EDX spectra shown in **(C)**, which allowed us to identify the background elements that were present in the cellular matrix.

TEM was used to visualize individual bacterial cells that had been isolated from Pavilion Lake. These MTB were found to be spirillum-shaped, 2.9 (±0.6) μ m long, 0.34 (±0.02) μ m wide (*n* = 7), containing a single flagellum (Figure 3A). The magnetosomes contained crystals of iron and oxygen indicating Fe_3_O_4_ (Figure 3B). Background spectra collected from the cellular matrix contained additional elements such as Mg, Na, Si, P, S, Cl, and K (Figure 3C). The Cu peaks that are seen in Figures 3B,C originated from the grid support used to mount the samples for TEM. The other elements (e.g., Na, Mg, P, K, S, Cl) observed in the EDX spectrum are from the cellular matrix (e.g., proteins, cytoplasm) and the dried fluid from the collection process (e.g., Al, Na, Si, Cl).

**Figure 4:**
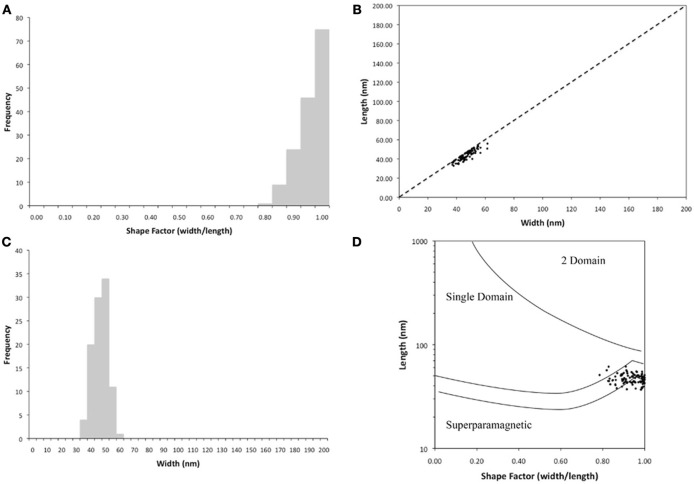
**Characterization of the magnetosomes contained within the magnetotactic bacteria that were isolated from Pavilion Lake. (A)** Frequency of the shape factor demonstrating that the crystals are nearly equal in width and length. **(B)** Width measurements of the magnetosomes demonstrate narrow size distribution of the crystals of magnetite. **(C)** Length and width of the magnetite crystals. **(D)** The sizes of the magnetic nanocrystals in our specimens fall within the single domain size range based on (Butler and Banerjee, 1975).

The Fe_3_O_4_ crystals in the cells averaged 47 (±4) nm long and 44 (±5) nm wide. The size range was 37–62 and 33–56 nm, respectively, for length and width (*n* = 155, Figure 4) with an average number of 19 crystals per cell (*n* = 7). The magnetite crystals were nearly the same size in length and width (Figure 4C) and most had a shape factor around 0.9 (±0.05) (Figure 4A) indicating that the magnetite crystals are single domain magnetite crystals (Figure 4D).

## Discussion

This study expands the range of habitats for MTB to include a freshwater ecosystem that contains microbialites. The MTB isolated from Pavilion Lake were spirillum-shaped bacteria from the *Alphaproteobacteria* that are closely related to *Magnetospirillum* (Figure 2). The bacteria were 2.9 μm long and 0.3 μm wide and contained a single chain of magnetosomes with an average of 19 cuboidal-shaped crystals per cell. The EDX data (Figures 3B,C) clearly demonstrated that the nanometer-sized minerals were composed of iron and oxygen, as expected for Fe_3_O_4_. The magnetite nanominerals were approximately 45 nm in diameter (Figure 4).

These measurements for MTB isolated from Pavilion Lake (Figure 3A) are similar to other freshwater *Magnetospirillum* bacteria, which have single chains of 15 or more magnetosomes with cuboctahedral shaped magnetite ranging from 40 to 50 nm (Isambert et al., 2007; Faivre and Schüler, 2008; Baumgartner and Faivre, 2011). In addition, the MTB that we isolated from Pavilion Lake had a polar flagellum, similar shape, and similar size as other freshwater *Magnetospirillum* (Figure 3A).

*Magnetospirillum* are ubiquitous in freshwater habitats and are one of the most common magnetotactic forms of *Alphaproteobacteria* (Spring and Bazylinski, 2006; Amann et al., 2007). Environmental parameters have been shown to affect the morphology of magnetosome crystals in culture (Spring and Schleifer, 1995; Faivre et al., 2008; Li and Pan, 2012) as well as the mineral composition both in the environment and in culture (Bazylinski et al., 1995; Simmons and Edwards, 2007; Lef èvre et al., 2011). The chemical composition and crystal morphology of magnetite crystals in our specimens were comparable to other *Magnetospirillum* described in the literature. Therefore, it appears that the unique freshwater environment of Pavilion Lake does not affect the “typical” magnetite crystals in magnetosomes of MTB. Perhaps a more detailed chemical and redox profiling of the microbialites as well as the water and sediment might reveal what environmental parameters are important in this regard.

Based on morphological and phylogenetic analysis of the bacteria from Pavilion Lake we were able to isolate one type of MTB (*Alphaproteobacteria*, Figure 2). None were found that belonged to the *Deltaproteobacteria* class or *Gammaproteobacteria* class or the *Nitrospirae* phylum. While it is possible that the collection and enrichment protocol used here selected for specific MTB species (Lin et al., 2008), this seems doubtful given the numerous studies where these types of techniques have been used to successfully retrieve MTB from the *Nitrospirae* phylum as well as the *Proteobacteria* (Lefèvre et al., 2011). In addition, MTB of the *Nitrospirae* and the *Deltaproteobacteria* are only known to biomineralize bullet-shaped magnetite magnetosomes (Lef èvre et al., 2013) and we did not observe these using electron microscopy. Thus, it seems more likely that *Nitrospirae* MTB were not present. Of course, one should be mindful of the fact that bacteria were not enriched from our lake samples until several weeks after collection. Some work has shown that this could have selected for one dominant type of MTB during the incubation period in our laboratory (Vali et al., 1987; Flies et al., 2005).

The samples analyzed in this paper confirm that MTB are present in the shallow water along the shoreline of Pavilion Lake. The next logical step is to use Scuba to access deeper parts of Pavilion Lake to determine whether MTB are also present in the microbialites. The presence of MTB in the microbialites in Pavilion Lake would have important implications for finding microfossils in these structures, especially when one considers that magnetite is known to serve as a robust biomarker in magnetofossils (Kirschvink and Chang, 1984; Chang and Kirschvink, 1989; Kobayashi et al., 2006; Kopp et al., 2006; Kopp and Kirschvink, 2008; Jimenez-Lopez et al., 2010; Pósfai et al., 2013). By determining whether magnetofossils and what magnetosome magnetite crystal morphologies are present in the microbialites, much important geologic, paleogeologic and geochemical information might be obtained. For example, it might be ascertained whether MTB played a significant role in the geochemical formation of the microbialites. By knowing what types of magnetofossil magnetite crystals are present, we may also be able to determine which phylogenetic and metabolic types of MTB (Lef èvre et al., 2013; Pósfai et al., 2013) are currently present as well as those associated with the microbialites in the past and when these associations existed. Finally, the presence of magnetofossils in the microbialites, together with geochemical studies, one might be able to determine specific chemical/redox conditions under which magnetite magnetofossils are chemically stable and persist in such environments.

### Conflict of interest statement

The authors declare that the research was conducted in the absence of any commercial or financial relationships that could be construed as a potential conflict of interest.
